# Unstable Hemoglobin, a Rare but Significant Cause of Hemolytic Anemia: Recognition of Peripheral Smear Findings Is Crucial for Diagnosis

**DOI:** 10.1111/ijlh.70043

**Published:** 2025-12-15

**Authors:** Ryan C. Shean, Archana Agarwal, Anton V. Rets

**Affiliations:** ^1^ ARUP Laboratories Salt Lake City Utah USA; ^2^ University of Utah Department of Pathology Salt Lake City Utah USA

**Keywords:** blood smear, Hb Köln, hemolytic anemia, HPLC, unstable hemoglobin

A 10‐year‐old male hospitalized for abdominal pain and jaundice was referred for hereditary hemolytic anemia workup. At admission, complete blood count (CBC) revealed severe normocytic normochromic anemia (hemoglobin (Hb) < 50 g/L, red blood cell (RBC) count < 2 × 10^12^/L), with normal mean corpuscular Hb concentration (MCHC) and mean corpuscular volume (MCV).

Pre‐transfusion peripheral smear (Figure 1, top panel) demonstrated normocytic normochromic erythrocytes with moderate anisopoikilocytosis, polychromasia, frequent bite cells (asterisk), echinocytes (x), and blister cells (arrow). Capillary electrophoresis (CE) and high‐performance liquid chromatography (HPLC) (Bio‐Rad) revealed abnormal patterns of Hb fractionation (bottom panel). CE showed mildly decreased Hb A (84%) with an unknown peak in the Z8 region (asterisk, 12%), suggesting a variant Hb. HPLC showed a slightly asymmetric “Hb A” peak (red dotted circle) of 78% and an abnormal peak of 12% at the retention time of 4.89 min (arrow). RBC osmotic fragility was increased, but Band 3 protein by flow cytometry and enzyme activities of pyruvate kinase and glucose‐6‐phosphate dehydrogenase (G6PD) were normal. Molecular testing demonstrated a heterozygous *HBB* variant c.295G>A; p.Val99Met, also known as Hb Köln.

**FIGURE 1:**
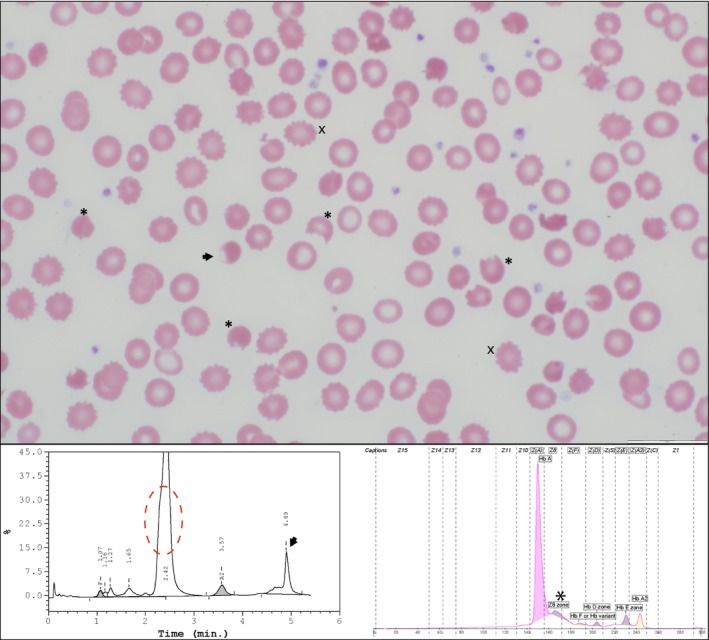
Panel 1. Peripheral blood smear (Wright‐Giemsa stain, 100×): Numerous “bite cells” (asterisk), a “blister cell” (arrow), and echinocytes (x). Panel 2. High‐performance liquid chromatography (HPLC) and capillary electrophoresis (CE) in a patient with Hb Köln trait: Coelution of Hb A and Hb Köln on HPLC results in asymmetry of the “Hb A” peak (red dotted circle). On CE Hb Köln is noted in the Z8 zone (asterisk). Additionally, as Hb Köln is unstable an additional peak formed by denatured Hb Köln may be noted at 4.89 min (arrow).

Hb Köln is caused by a point mutation in *HBB* gene resulting in an abnormal beta‐globin chain with decreased stability [1]. The valine to methionine substitution in Hb Köln lies in a key binding region for beta‐globin folding and heme binding. The mutation leads to a hemoglobin tetramer with abnormally high oxygen affinity [2] and a tendency to denature.

When Hb Köln (and other unstable Hb) undergo oxidative damage, the denatured hemoglobin precipitates as intracellular inclusions called Heinz bodies. Heinz bodies are not visible on Wright‐Giemsa stains but may be viewed with supravital stains. Additionally, they can attach to the RBC membrane, forming “blister cells”. The precipitated globin in the Heinz bodies makes the RBCs less deformable, and thus the splenic macrophages remove these inclusions, causing the characteristic “bite cells” and ultimately extravascular hemolysis. These cells are not specific for unstable hemoglobin and can be seen in G6PD deficiency, oxidative drug‐induced hemolysis, and thalassemias.

The creation of bite and blister cells as well as extravascular hemolysis leads to increased variation in size and shape of erythrocytes (anisopoikilocytosis). Although polychromasia is not noted in this photomicrograph field, increased production of immature erythrocytes may be seen as a compensatory response to anemia.

Usually, because there is no charge or affinity change of the Hb Köln mutation, the variant hemoglobin elutes with Hb A and is frequently covered by the A peak, which can make diagnosis difficult. However, the asymmetric peak in the Hb A region (red circle) should raise suspicion. In our experience, in cases with Hb Köln and no normal Hb A, Hb Köln elutes on HPLC at 2.29 min. Additionally, the denatured Hb Köln can precipitate and be seen as an abnormal peak at ~5 min (black arrow).

Despite its overall rarity, Hb Köln is one of the most encountered unstable Hb variants. Recognizing the characteristic smear findings and biochemical profile of unstable hemoglobins like Hb Köln is crucial to avoid misdiagnosis and guide appropriate management [3].

## Author Contributions

All authors created and edited the manuscript.

## Funding

The authors have nothing to report.

## Conflicts of Interest

The authors declare no conflicts of interest.

## Data Availability

The data that support the findings of this study are available from the corresponding author upon reasonable request.
